# PReS-FINAL-2086: In vitro investigation of the sustained therapeutic effect of etanercept loaded microspheres on human rheumatoid arthritis fibroblast-like synoviocytes

**DOI:** 10.1186/1546-0096-11-S2-P98

**Published:** 2013-12-05

**Authors:** O Erdemli, S Ozen, C Kocaefe, A Usanmaz, ED Batu, B Atilla, D Keskin, A Tezcaner

**Affiliations:** 1 Engineering Sciences, Middle East Technical University, Ankara, Turkey; 2 Paediatric Rheumatology, Hacettepe University, Ankara, Turkey; 3 Medical Biology, Hacettepe University, Ankara, Turkey; 4 Chemistry, Middle East Technical University, Ankara, Turkey; 5 Orthopedics and Traumatology, Hacettepe University, Ankara, Turkey; 6 Biomaterials and Tissue Engineering Center of Excellence, Middle East Technical University, Ankara, Turkey

## Introduction

Anti-TNFα treatment has become an important part of our systemic treatment in chronic inflammatory arthritis. Local application to the joint produces transient clinical improvement since the drug quickly leaves the joint.

## Objectives

We aimed to develop a sustained release system for an anti-TNFα drug in treatment of chronic inflammatory arthritis. A novel form of intra-articularly injectable etanercept (ETN) loaded poly(ε-caprolactone) (PCL) or methoxy poly(ethylene glycol)-poly(ε-caprolactone)-methoxy poly(ethylene glycol) (MPEG-PCL-MPEG) microspheres (patent pending) were prepared to provide long term controlled release of ETN with a sustained anti-inflammatory effect as a local treatment approach.

## Methods

Size, surface morphology, encapsulation efficiency, and in vitro release profiles of γ-sterilized microspheres loaded with ETN were determined. Treatment efficacies of free and microsphere loaded ETN were evaluated by determining changes in cell number and viability of fibroblast-like synoviocytes (FLS), in protein levels of pro-inflammatory cytokines (TNFα, IL-6, IFNγ, IL-17) and MMPs (MMP-3 and MMP-13) and in mRNA expressions of TNFα, IL-6, MMP-3 and MMP-13.

## Results

Microspheres possessed a rough surface and had a mean particle size around 5 μm. MPEG-PCL-MPEG microspheres had higher drug encapsulation efficiency than PCL microspheres. Total amounts of biologically active ETN released from MPEG-PCL-MPEG microspheres were significantly higher than that from PCL microspheres at each time point during the four weeks study period. FLS viability significantly decreased in the free drug group at first week whereas no significant decrease was observed in microsphere groups. ETN loaded microspheres significantly decreased the levels of pro-inflammatory cytokines TNFα, IL-6, IFNγ, IL-17 and MMPs in FLSs. However, there were no significant variations in the gene expressions of pro-inflammatory cytokines and MMPs among groups.

## Conclusion

ETN loaded microspheres provide a sustained release, which resulted with a significant decrease in pro-inflammatory cytokines and MMPs levels. This study showed that MPEG-PCL-MPEG and PCL microspheres are promising and safe systems for an effective local treatment approach in chronic inflammatory arthritis.

*This study is a part of patent pending invention (PCT No: PCT/TR2012/000148)*.

## Disclosure of interest

O. Erdemli Shareholder of: Patent Pending (PCT No: PCT/TR2012/000148), Grant/Research Support from: The Scientific and Technological Research Council of Turkey (Grant no: 109S104), S. Ozen Shareholder of: Patent Pending (PCT No: PCT/TR2012/000148), C. Kocaefe Shareholder of: Patent Pending (PCT No: PCT/TR2012/000148), A. Usanmaz Shareholder of: Patent Pending (PCT No: PCT/TR2012/000148), E. Batu: None declared, B. Atilla Shareholder of: Patent Pending (PCT No: PCT/TR2012/000148), D. Keskin Shareholder of: Patent Pending (PCT No: PCT/TR2012/000148), A. Tezcaner Shareholder of: Patent Pending (PCT No: PCT/TR2012/000148).

